# Social impact in social media: A new method to evaluate the social impact of research

**DOI:** 10.1371/journal.pone.0203117

**Published:** 2018-08-29

**Authors:** Cristina M. Pulido, Gisela Redondo-Sama, Teresa Sordé-Martí, Ramon Flecha

**Affiliations:** 1 Department of Journalism and Communication Studies, Universitat Autonoma de Barcelona, Barcelona, Spain; 2 Department of Psychology and Sociology, Universidad de Zaragoza, Zaragoza, Spain; 3 Department of Sociology, Universitat Autonoma de Barcelona, Barcelona, Spain; 4 Department of Sociology, Universitat de Barcelona (UB), Barcelona, Spain; Institut Català de Paleoecologia Humana i Evolució Social (IPHES), SPAIN

## Abstract

The social impact of research has usually been analysed through the scientific outcomes produced under the auspices of the research. The growth of scholarly content in social media and the use of altmetrics by researchers to track their work facilitate the advancement in evaluating the impact of research. However, there is a gap in the identification of evidence of the social impact in terms of what citizens are sharing on their social media platforms. This article applies a social impact in social media methodology (SISM) to identify quantitative and qualitative evidence of the potential or real social impact of research shared on social media, specifically on Twitter and Facebook. We define the social impact coverage ratio (SICOR) to identify the percentage of tweets and Facebook posts providing information about potential or actual social impact in relation to the total amount of social media data found related to specific research projects. We selected 10 projects in different fields of knowledge to calculate the SICOR, and the results indicate that 0.43% of the tweets and Facebook posts collected provide linkages with information about social impact. However, our analysis indicates that some projects have a high percentage (4.98%) and others have no evidence of social impact shared in social media. Examples of quantitative and qualitative evidence of social impact are provided to illustrate these results. A general finding is that novel evidences of social impact of research can be found in social media, becoming relevant platforms for scientists to spread quantitative and qualitative evidence of social impact in social media to capture the interest of citizens. Thus, social media users are showed to be intermediaries making visible and assessing evidence of social impact.

## Introduction

The social impact of research is at the core of some of the debates influencing how scientists develop their studies and how useful results for citizens and societies may be obtained. Concrete strategies to achieve social impact in particular research projects are related to a broader understanding of the role of science in contemporary society. There is a need to explore dialogues between science and society not only to communicate and disseminate science but also to achieve social improvements generated by science. Thus, the social impact of research emerges as an increasing concern within the scientific community [1]. As Bornmann [2] said, the assessment of this type of impact is badly needed and is more difficult than the measurement of scientific impact; for this reason, it is urgent to advance in the methodologies and approaches to measuring the social impact of research.

Several authors have approached the conceptualization of social impact, observing a lack of generally accepted conceptual and instrumental frameworks [3]. It is common to find a wide range of topics included in the contributions about social impact. In their analysis of the policies affecting land use, Hemling et al. [4] considered various domains in social impact, for instance, agricultural employment or health risk. Moving to the field of flora and fauna, Wilder and Walpole [5] studied the social impact of conservation projects, focusing on qualitative stories that provided information about changes in attitudes, behaviour, wellbeing and livelihoods. In an extensive study by Godin and Dore [6], the authors provided an overview and framework for the assessment of the contribution of science to society. They identified indicators of the impact of science, mentioning some of the most relevant weaknesses and developing a typology of impact that includes eleven dimensions, with one of them being the impact on society. The subdimensions of the impact of science on society focus on individuals (wellbeing and quality of life, social implication and practices) and organizations (speeches, interventions and actions). For the authors, social impact “refers to the impact knowledge has on welfare, and on the behaviours, practices and activities of people and groups” (p. 7).

In addition, the terms “social impact” and “societal impact” are sometimes used interchangeably. For instance, Bornmann [2] said that due to the difficulty of distinguishing social benefits from the superior term of societal benefits, “in much literature the term ‘social impact’ is used instead of ‘societal impact’”(p. 218). However, in other cases, the distinction is made [3], as in the present research. Similar to the definition used by the European Commission [7], social impact is used to refer to economic impact, societal impact, environmental impact and, additionally, human rights impact. Therefore, we use the term social impact as the broader concept that includes social improvements in all the above mentioned areas obtained from the transference of research results and representing positive steps towards the fulfilment of those officially defined social goals, including the UN Sustainable Development Goals, the EU 2020 Agenda, or similar official targets. For instance, the Europe 2020 strategy defines five priority targets with concrete indicators (employment, research and development, climate change and energy, education and poverty and social exclusion) [8], and we consider the targets addressed by objectives defined in the specific call that funds the research project.

This understanding of the social impact of research is connected to the creation of the Social Impact Open Repository (SIOR), which constitutes the first open repository worldwide that displays, cites and stores the social impact of research results [9]. The SIOR has linked to ORCID and Wikipedia to allow the synergies of spreading information about the social impact of research through diverse channels and audiences. It is relevant to mention that currently, SIOR includes evidence of real social impact, which implies that the research results have led to actual improvements in society. However, it is common to find evidence of potential social impact in research projects. The potential social impact implies that in the development of the research, there has been some evidence of the effectiveness of the research results in terms of social impact, but the results have not yet been transferred.

Additionally, a common confusion is found among the uses of dissemination, transference (policy impact) and social impact. While dissemination means to disseminate the knowledge created by research to citizens, companies and institutions, transference refers to the use of this knowledge by these different actors (or others), and finally, as already mentioned, social impact refers to the actual improvements resulting from the use of this knowledge in relation to the goals motivating the research project (such as the United Nations Sustainable Development Goals). In the present research [3], it is argued that “social impact can be understood as the culmination of the prior three stages of the research” (p.3). Therefore, this study builds on previous contributions measuring the dissemination and transference of research and goes beyond to propose a novel methodological approach to track social impact evidences.

In fact, the contribution that we develop in this article is based on the creation of a new method to evaluate the evidence of social impact shared in social media. The evaluation proposed is to measure the social impact coverage ratio (SICOR), focusing on the presence of evidence of social impact shared in social media. Then, the article first presents some of the contributions from the literature review focused on the research on social media as a source for obtaining key data for monitoring or evaluating different research purposes. Second, the SISM (social impact through social media) methodology[10] developed is introduced in detail. This methodology identifies quantitative and qualitative evidence of the social impact of the research shared on social media, specifically on Twitter and Facebook, and defines the SICOR, the social impact coverage ratio. Next, the results are discussed, and lastly, the main conclusions and further steps are presented.

## Literature review

Social media research includes the analysis of citizens’ voices on a wide range of topics [11]. According to quantitative data from April 2017 published by Statista [12], Twitter and Facebook are included in the top ten leading social networks worldwide, as ranked by the number of active users. Facebook is at the top of the list, with 1,968 million active users, and Twitter ranks 10^th^, with 319 million active users. Between them are the following social networks: WhatsApp, YouTube, Facebook Messenger, WeChat, QQ, Instagram,Qzone and Tumblr. If we look at altmetrics, the tracking of social networks for mentions of research outputs includes Facebook, Twitter, Google+,LinkedIn, Sina Weibo and Pinterest. The social networks common to both sources are Facebook and Twitter. These are also popular platforms that have a relevant coverage of scientific content and easy access to data, and therefore, the research projects selected here for application of the SISM methodology were chosen on these platforms.

Chew and Eysenbach [13] studied the presence of selected keywords in Twitter related to public health issues, particularly during the 2009 H1N1 pandemic, identifying the potential for health authorities to use social media to respond to the concerns and needs of society. Crooks et al.[14] investigated Twitter activity in the context of a 5.8 magnitude earthquake in 2011 on the East Coast of the United States, concluding that social media content can be useful for event monitoring and can complement other sources of data to improve the understanding of people’s responses to such events. Conversations among young Canadians posted on Facebook and analysed by Martinello and Donelle [15] revealed housing and transportation as main environmental concerns, and the project *FoodRisc* examined the role of social media to illustrate consumers’ quick responses during food crisis situations [16]. These types of contributions illustrate that social media research implies the understanding of citizens’ concerns in different fields, including in relation to science.

Research on the synergies between science and citizens has increased over the years, according to Fresco [17], and there is a growing interest among researchers and funding agencies in how to facilitate communication channels to spread scientific results. For instance, in 1998, Lubchenco [18] advocated for a social contract that “represents a commitment on the part of all scientists to devote their energies and talents to the most pressing problems of the day, in proportion to their importance, in exchange for public funding”(p.491).

In this framework, the recent debates on how to increase the impact of research have acquired relevance in all fields of knowledge, and major developments address the methods for measuring it. As highlighted by Feng Xia et al. [19], social media constitute an emerging approach to evaluating the impact of scholarly publications, and it is relevant to consider the influence of the journal, discipline, publication year and user type. The authors revealed that people’s concerns differ by discipline and observed more interest in papers related to everyday life, biology, and earth and environmental sciences. In the field of biomedical sciences, Haustein et al. [20] analysed the dissemination of journal articles on Twitter to explore the correlations between tweets and citations and proposed a framework to evaluate social media-based metrics. In fact, different studies address the relationship between the presence of articles on social networks and citations [21]. Bornmann [22] conducted a case study using a sample of 1,082 PLOS journal articles recommended in F1000 to explore the usefulness of altmetrics for measuring the broader impact of research. The author presents evidence about Facebook and Twitter as social networks that may indicate which papers in the biomedical sciences can be of interest to broader audiences, not just to specialists in the area. One aspect of particular interest resulting from this contribution is the potential to use altmetrics to measure the broader impacts of research, including the societal impact. However, most of the studies investigating social or societal impact lack a conceptualization underlying its measurement.

To the best of our knowledge, the assessment of social impact in social media (SISM) has developed according to this gap. At the core of this study, we present and discuss the results obtained through the application of the SICOR (social impact coverage ratio) with examples of evidence of social impact shared in social media, particularly on Twitter and Facebook, and the implications for further research.

Following these previous contributions, our research questions were as follows: Is there evidence of social impact of research shared by citizens in social media? If so, is there quantitative or qualitative evidence? How can social media contribute to identifying the social impact of research?

## Methods and data presentation

A group of new methodologies related to the analysis of online data has recently emerged. One of these emerging methodologies is social media analytics [23], which was initially used most in the marketing research field but also came to be used in other domains due to the multiple possibilities opened up by the availability and richness of the data for different research purposes. Likewise, the concern of how to evaluate the social impact of research as well as the development of methodologies for addressing this concern has occupied central attention. The development of SISM (Social Impact in Social Media) and the application of the SICOR (Social Impact Coverage Ratio) is a contribution to advancement in the evaluation of the social impact of research through the analysis of the social media selected (in this case, Twitter and Facebook). Thus, SISM is novel in both social media analytics and among the methodologies used to evaluate the social impact of research. This development has been made under IMPACT-EV, a research project funded under the Framework Program FP7 of the Directorate-General for Research and Innovation of the European Commission. The main difference from other methodologies for measuring the social impact of research is the disentanglement between dissemination and social impact. While altmetrics is aimed at measuring research results disseminated beyond academic and specialized spheres, SISM contribute to advancing this measurement by shedding light on to what extent evidence of the social impact of research is found in social media data. This involves the need to differentiate between tweets or Facebook posts (Fb/posts) used to disseminate research findings from those used to share the social impact of research. We focus on the latter, investigating whether there is evidence of social impact, including both potential and real social impact. In fact, the question is whether research contributes and/or has the potential to contribute to improve the society or living conditions considering one of these goals defined. What is the evidence? Next, we detail the application of the methodology.

### Data collection

To develop this study, the first step was to select research projects with social media data to be analysed. The selection of research projects for application of the SISM methodology was performed according to three criteria.

Criteria 1. Selection of success projects in FP7. The projects were success stories of the 7^th^ Framework Programme (FP7) highlighted by the European Commission [24] in the fields of knowledge of medicine, public health, biology and genomics. The FP7 published calls for project proposals from 2007 to 2013. This implies that most of the projects funded in the last period of the FP7 (2012 and 2013) are finalized or in the last phase of implementation.

Criteria 2. Period of implementation. We selected projects in the 2012–2013 period because they combine recent research results with higher possibilities of having Twitter and Facebook accounts compared with projects of previous years, as the presence of social accounts in research increased over this period.

Criteria 3. Twitter and Facebook accounts. It was crucial that the selected projects had active Twitter and Facebook accounts.

Table 1 summarizes the criteria and the final number of projects identified. As shown, 10 projects met the defined criteria. Projects in medical research and public health had higher presence.

**Table 1:** Selection criteria and number of projects.

Field of Knowledge	Criteria 1. Success stories FP7	Criteria 2. Starting year 2012 & 2013	Criteria 3. Twitter and Facebook
**Medical research**	98	11	3
**Public Health**	37	11	4
**Genomics**	14	2	1
**Biology**	9	2	2
**TOTAL**	158	26	**10**

After the selection of projects, we defined the timeframe of social media data extraction on Twitter and Facebook from the starting date of the project until the day of the search, as presented in Table 2.

**Table 2:** Timeframe for the extraction of Twitter and Facebook data.

Project	Period	Months
Project 1	From 2012-11-01 to 2017-04-25	54.53
Project 2	From 2012-11-01 to 2017-04-25	54.53
Project 3	From 2013-01-01 to 2017-04-25	52.50
Project 4	From 2013-10-01 to 2017-04-25	43.40
Project 5	From 2013-11-01 to 2017-04-25	42.37
Project 6	From 2013-02-01 to 2017-04-25	51.47
Project 7	From 2013-11-01 to 2017-04-25	42.37
Project 8	From 2012-11-01 to 2017-04-25	54.54
Project 9	From 2012-11-01 to 2017-04-25	54.54
Project 10	From 2012-08-01 to 2017-04-25	57.60

The second step was to define the search strategies for extracting social media data related to the research projects selected. In this line, we defined three search strategies.

Strategy 1. To extract messages published on the Twitter account and the Facebook page of the selected projects. We listed the Twitter accounts and Facebook pages related to each project in order to look at the available information. In this case, it is important to clarify that the tweets published under the corresponding Twitter project account are original tweets or retweets made from this account. It is relevant to mention that in one case, the Twitter account and Facebook page were linked to the website of the research group leading the project. In this case, we selected tweets and Facebook posts related to the project. For instance, in the case of the Twitter account, the research group created a specific hashtag to publish messages related to the project; therefore, we selected only the tweets published under this hashtag. In the analysis, we prioritized the analysis of the tweets and Facebook posts that received some type of interaction (likes, retweets or shares) because such interaction is a proxy for citizens’ interest. In doing so, we used the R program and NVivoto extract the data and proceed with the analysis. Once we obtained the data from Twitter and Facebook, we were able to have an overview of the information to be further analysed, as shown in Table 3.

**Table 3:** Tweets and Facebook posts per project obtained–Strategy 1.

Project	Tweets	Facebook posts
Project 1	952	585
Project 2	403	423
Project 3	896	396
Project 4	21	41
Project 5	410	16
Project 6	124	74
Project 7	148	64
Project 8	56	236
Project 9	55	43
Project 10	106	47
TOTAL	3,171	1,925

We focused the second and third strategies on Twitter data. In both strategies, we extracted Twitter data directly from the Twitter Advanced Search tool, as the API connected to NVivo and the R program covers only a specific period of time limited to 7/9 days. Therefore, the use of the Twitter Advanced Search tool made it possible to obtain historic data without a period limitation. We downloaded the results in PDF and then uploaded them to NVivo.

Strategy 2. To use the project acronym combined with other keywords, such as FP7 or EU. This strategy made it possible to obtain tweets mentioning the project. Table 4 presents the number of tweets obtained with this strategy.

**Table 4:** Tweets obtained per project–Strategy 2.

Project	Tweets
Project 1	10
Project 2	0
Project 3	2
Project 4	5
Project 5	4
Project 6	175
Project 7	4
Project 8	5
Project 9	4
Project 10	17
TOTAL	226

Strategy 3. To use searchable research results of projects to obtain Twitter data. We defined a list of research results, one for each project, and converted them into keywords. We selected one searchable keyword for each project from its website or other relevant sources, for instance, the brief presentations prepared by the European Commission and published in CORDIS. Once we had the searchable research results, we used the Twitter Advanced Search tool to obtain tweets, as presented in Table 5.

**Table 5:** List of searchable research results–Strategy 3.

Project	Searchable Research result	Tweets
Project 1	MACSQuant® Tyto	3
Project 2	Prototype screening tests for pre-eclampsia	0
Project 3	Early Life Exposome	5
Project 4	Splendid system	0
Project 5	EuroFIT programme	4
Project 6	Fishchoice tool	3
Project 7	Vitamin D-enhanced eggs	5
Project 8	Developakure clinical trials	4
Project 9	Precision Livestock Farming Applications	3
Project 10	NOSHAN technologies	1
TOTAL		28

The sum of the data obtained from these three strategies allowed us to obtain a total of 3,425 tweets and 1,925 posts on public Facebook pages. Table 6 presents a summary of the results.

**Table 6:** Summary of the Twitter and Facebook data collected.

Project	Tweets	Facebook posts
Project 1	965	585
Project 2	403	423
Project 3	903	396
Project 4	26	41
Project 5	418	16
Project 6	302	74
Project 7	157	64
Project 8	65	236
Project 9	62	43
Project 10	124	47
TOTAL	3,425	1,925

We imported the data obtained from the three search strategies into NVivo to analyse. Next, we select tweets and Facebook posts providing linkages with quantitative or qualitative evidence of social impact, and we complied with the terms of service for the social media from which the data were collected. By quantitative and qualitative evidence, we mean data or information that shows how the implementation of research results has led to improvements towards the fulfilment of the objectives defined in the EU2020 strategy of the European Commission or other official targets. For instance, in the case of quantitative evidence, we searched tweets and Facebook posts providing linkages with quantitative information about improvements obtained through the implementation of the research results of the project. In relation to qualitative evidence, for example, we searched for testimonies that show a positive evaluation of the improvement due to the implementation of research results. In relation to this step, it is important to highlight that social media users are intermediaries making visible evidence of social impact. Users often share evidence, sometimes sharing a link to an external resource (e.g., a video, an official report, a scientific article, news published on media). We identified evidence of social impact in these sources.

### Data analysis

We analysed all tweets and Facebook posts collected (3,425 tweets and 1,925 Facebook posts) to calculate the ratio of social media data with evidence of social impact in relation to the total amount of social media data extracted from the research projects selected. The aim was to answer the question whether or not there is evidence of social impact shared by citizens in social media. Once we had the tweets and Facebook posts selected for each project, we identified the number of tweets and Facebook posts responding or not to the criteria of presenting evidence of the social impact of research. In the final step of this search, we defined a ratio of coverage adapted to this calculation called the SICOR, the social impact coverage ratio:

$$SICOR=\frac{\sum_{i=1}^{n}\gamma_{i}}{\sum_{i=1}^{n}T_{i}}=\frac{\gamma_{1}+\gamma_{2}+\ldots +\gamma_{n}}{T_{1}+T_{2}+\ldots +T_{n}}$$

where:

***γ_i_*** is the total number of messages obtained about project *i* with evidence of social impact on social media platforms (Twitter, Facebook, Instagram, etc.);

***T***_*i*_ is the total number of messages from project *i*on social media platforms (Twitter, Facebook, Instagram, etc.); and

**n** is the number of projects selected.

The result is expressed in percentages. In this paper, we use the SICOR for Twitter and Facebook thus:

γ∈{Tw,Fb}

and

T∈{Tw,Fb}.

### Analytical categories and codebook

The researchers who carried out the analysis of the social media data collected are specialists in the social impact of research and research on social media. Before conducting the full analysis, two aspects were guaranteed. First, how to identify evidence of social impact relating to the targets defined by the EU2020 strategy or to specific goals defined by the call addressed was clarified. Second, we held a pilot to test the methodology with one research project that we know has led to considerable social impact, which allowed us to clarify whether or not it was possible to detect evidence of social impact shared in social media. Once the pilot showed positive results, the next step was to extend the analysis to another set of projects and finally to the whole sample. The construction of the analytical categories was defined a priori, revised accordingly and lastly applied to the full sample.

Different observations should be made. First, in this previous analysis, we found that the tweets and Facebook users play a key role as “intermediaries,” serving as bridges between the larger public and the evidence of social impact. Social media users usually share a quote or paragraph introducing evidence of social impact and/or link to an external resource, for instance, a video, official report, scientific article, news story published on media, etc., where evidence of the social impact is available. This fact has implications for our study, as our unit of analysis is all the information included in the tweets or Facebook posts. This means that our analysis reaches the external resources linked to find evidence of social impact, and for this reason, we defined tweets or Facebook posts providing linkages with information about social impact.

Second, the other important aspect is the analysis of the users’ profile descriptions, which requires much more development in future research given the existing limitations. For instance, some profiles are users’ restricted due to privacy reasons, so the information is not available; other accounts have only the name of the user with no description of their profile available. Therefore, we gave priority to the identification of evidence of social impact including whether a post obtained interaction (retweets, likes or shares) or was published on accounts other than that of the research project itself. In the case of the profile analysis, we added only an exploratory preliminary result because this requires further development. Considering all these previous details, the codebook (see Table 7) that we present as follows is a result of this previous research.

**Table 7:** Codebook of SISM.

CODE	Element	Definition
ESISM	Evidence of social impact shared in social media	Evidence of social impact is a research result that contributes to the achievement of a particular objective of the society defined by the corresponding institution,in this case, one of the targets addressed in the EU2020 strategy or the target addressed in the call of the funding project. Evidence can be of potential or already achieved social impact.
QUALESISM	Qualitative evidence of social impact	The evidence provided gives qualitative information about improvements obtained through the implementation of the research results of the project linked to the one of the targets of the EU2020 strategy or the target addressed in the call of the funding project. Evidence can be of potential or already achieved social impact.
QUANESISM	Quantitative evidence of social impact	The evidence provided gives quantitative information about improvements obtained through the implementation of the research results of the project linked to the one of the targets of the EU2020 strategy or the target addressed in the call of the funding project. Evidence can be of potential or already achieved social impact.
INTER	Interaction of the tweet or Fb post	The tweet or post has been shared, liked retweeted or published by an account other than the project account itself.
PROF D	Diverse profiles	Diverse profiles of citizens have interacted with the tweet or Fb post.

### How to analyse Twitter and Facebook data

To illustrate how we analysed data from Twitter and Facebook, we provide one example of each type of evidence of social impact defined, considering both real and potential social impact, with the type of interaction obtained and the profiles of those who have interacted.

QUANESISM. Tweet by ZeroHunger Challenge @ZeroHunger published on 3 May 2016. Text: How re-using food waste for animal feed cuts carbon emissions.-NOSHAN project hubs.ly/H02SmrP0. 7 retweets and 5 likes.

The unit of analysis is all the content of the tweet, including the external link. If we limited our analysis to the tweet itself, it would not be evidence. Examining the external link is necessary to find whether there is evidence of social impact. The aim of this project was to investigate the process and technologies needed to use food waste for feed production at low cost, with low energy consumption and with a maximal evaluation of the starting wastes. This tweet provides a link to news published in the PHYS.org portal [25], which specializes in science news. The news story includes an interview with the main researcher that provides the following quotation with quantitative evidence:

'Our results demonstrated that with a NOSHAN 10 percent mix diet, for every kilogram of broiler chicken feed, carbon dioxide emissions were reduced by 0.3 kg compared to a non-food waste diet,' explains Montse Jorba, NOSHAN project coordinator. 'If 1 percent of total chicken broiler feed in Europe was switched to the 10 percent NOSHAN mix diet, the total amount of CO2 emissions avoided would be 0.62 million tons each year.'[25]

This quantitative evidence “a NOSHAN 10 percent mix diet, for every kilogram of broiler chicken feed, carbon dioxide emissions carbon dioxide emissions were reduced by 0.3 kg to a non-food waste diet” is linked directly with the Europe 2020 target of Climate Change & Energy, specifically with the target of reducing greenhouse gas emissions by 20% compared to the levels in 1990 [8]. The illustrative extrapolation the coordinator mentioned in the news is also an example of quantitative evidence, although is an extrapolation based on the specific research result.

This tweet was captured by the Acronym search strategy. It is a message tweeted by an account that is not related to the research project. The twitter account is that of the Zero Hunger Challenge movement, which supports the goals of the UN. The interaction obtained is 7 retweets and 5 likes. Regarding the profiles of those who retweeted and clicked “like”, there were activists, a journalist, an eco-friendly citizen, a global news service, restricted profiles (no information is available on those who have retweeted) and one account with no information in its profile.

The following example illustrates the analysis of QUALESISM: Tweet by @eurofitFP7 published on4 October 2016. Text: See our great new EuroFIT video on youtube! https://t.co/TocQwMiW3c 9 retweets and 5 likes.

The aim of this project is to improve health through the implementation of two novel technologies to achieve a healthier lifestyle. The tweet provides a link to a video on YouTube on the project’s results. In this video, we found qualitative evidence from people who tested the EuroFit programme; there are quotes from men who said that they have experienced improved health results using this method and that they are more aware of how to manage their health:

One end-user said: I have really amazing results from the start, because I managed to change a lot of things in my life. And other one: I was more conscious of what I ate, I was more conscious of taking more steps throughout the day and also standing up a little more. [26]

The research applies the well researched scientific evidence to the management of health issues in daily life. The video presents the research but also includes a section where end-users talk about the health improvements they experienced. The quotes extracted are some examples of the testimonies collected. All agree that they have improved their health and learned healthy habits for their daily lives. These are examples of qualitative evidence linked with the target of the call HEALTH.2013.3.3–1—Social innovation for health promotion [27] that has the objectives of reducing sedentary habits in the population and promoting healthy habits. This research contributes to this target, as we see in the video testimonies. Regarding the interaction obtained, this tweet achieved 9 retweets and 5 likes. In this case, the profiles of the interacting citizens show involvement in sport issues, including sport trainers, sport enthusiasts and some researchers.

To summarize the analysis, in Table 8 below, we provide a summary with examples illustrating the evidence found.

**Table 8:** Examples of tweets and Facebook posts with quantitative evidence of social impact.

Tweet/ Fb post	Project
Weekly consumption of 7 vitamin D-enhanced eggs has an important impact on winter vitamin D status in adults. http://www.greenmedinfo.com/article/weekly consumption-7-vitamin-d-enhanced-eggs-has-important-impact-winter-vitam	Project 7
How re-using food waste for animal feed cuts carbon emissions NOSHAN Project: http://hubs.ly/H02SmrP0	Project 10
Here's a HELIX publication for you! Assessment of metabolic phenotypic variability in children's urine using 1H NMR spectroscopy.—PubMed—NCBI https://www.ncbi.nlm.nih.gov/pmc/articles/PMC5395814/	Project 3

### Quantitative evidence of social impact in social media

There is a greater presence of tweets/Fb posts with quantitative evidence (14) than with qualitative evidence (9) in the total number of tweets/Fb posts identified with evidence of social impact. Most of the tweets/Fb posts with quantitative evidence of social impact are from scientific articles published in peer-reviewed international journals and show potential social impact. In Table 8, we introduce 3 examples of this type of tweets/Fb posts with quantitative evidence:

The first tweet with quantitative social impact selected is from project 7. The aim of this project was to provide high-quality scientific evidence for preventing vitamin D deficiency in European citizens. The tweet highlighted the main contribution of the published study, that is, “Weekly consumption of 7 vitamin D-enhanced eggs has an important impact on winter vitamin D status in adults” [28]. The quantitative evidence shared in social media was extracted from a news publication in a blog on health news. This blog collects scientific articles of research results. In this case, the blog disseminated the research result focused on how vitamin D-enhanced eggs improve vitamin D deficiency in wintertime, with the published results obtained by the research team of the project selected. The quantitative evidence illustrates that the group of adults who consumed vitamin D-enhanced eggs did not suffer from vitamin D deficiency, as opposed to the control group, which showed a significant decrease in vitamin D over the winter. The specific evidence is the following extracted from the article [28]:

With the use of a within-group analysis, it was shown that, although serum 25(OH) D in the control group significantly decreased over winter (mean ± SD: -6.4 ± 6.7 nmol/L; P = 0.001), there was no change in the 2 groups who consumed vitamin D-enhanced eggs (P>0.1 for both. (p. 629)

This evidence contributes to achievement of the target defined in the call addressed that is KBBE.2013.2.2–03—Food-based solutions for the eradication of vitamin D deficiency and health promotion throughout the life cycle [29]. The quantitative evidence shows how the consumption of vitamin D-enhanced eggs reduces vitamin D deficiency.

The second example of this table corresponds to the example of quantitative evidence of social impact provided in the previous section.

The third example is a Facebook post from project 3 that is also tweeted. Therefore, this evidence was published in both social media sources analysed. The aim of this project was to measure a range of chemical and physical environmental hazards in food, consumer products, water, air, noise, and the built environment in the pre- and postnatal early-life periods. This Facebook post and tweet links directly to a scientific article [30] that shows the precision of the spectroscopic platform:

Using 1H NMR spectroscopy we characterized short-term variability in urinary metabolites measured from 20 children aged 8–9 years old. Daily spot morning, night-time and pooled (50:50 morning and night-time) urine samples across six days (18 samples per child) were analysed, and 44 metabolites quantified. Intraclass correlation coefficients (ICC) and mixed effect models were applied to assess the reproducibility and biological variance of metabolic phenotypes. Excellent analytical reproducibility and precision was demonstrated for the 1H NMR spectroscopic platform (median CV 7.2%)^.^(p.1)

This evidence is linked to the target defined in the call “ENV.2012.6.4–3—Integrating environmental and health data to advance knowledge of the role of environment in human health and well-being in support of a European exposome initiative” [31]. The evidence provided shows how the project’s results have contributed to building technology for improving the data collection to advance in the knowledge of the role of the environment in human health, especially in early life. The interaction obtained is one retweet from a citizen from Nigeria interested in health issues, according to the information available in his profile.

### Qualitative evidence of social impact in social media

We found qualitative evidence of the social impact of different projects, as shown in Table 9. Similarly to the quantitative evidence, the qualitative cases also demonstrate potential social impact. The three examples provided have in common that they are tweets or Facebook posts that link to videos where the end users of the research project explain their improvements once they have implemented the research results.

**Table 9:** Examples of tweets and Facebook posts with qualitative evidence of social impact.

Tweet/ Fb post	Project
'#Tech trialled in fight against ticking #obesity timebomb' #H2O20 #SPLENDID project, by @euronews http://www.euronews.com/2016/06/17/technology-trialled-in-fight-against-ticking-timebomb-of-obesity/ … via @eu_ehealth	Project 4
EU-PLF and Fancom b.v. in the news again. This time in Euronews! http://www.euronews.com/2016/05/09/big-farmer-is-watching-surveillance-technology-monitors-animal-wellbeing	Project 9
See our great new EuroFIT video on youtube! https://www.youtube.com/watch?v=CHkbnD8IgZw&feature=youtu.be	Project 5

The first tweet with qualitative evidence selected is from project 4. The aim of this project is to produce a system that helps in the prevention of obesity and eating disorders, targeting young people and adults [32]. The twitter account that published this tweet is that of the Future and Emerging Technologies Programme of the European Commission, and a link to a Euronews video is provided. This video shows how the patients using the technology developed in the research achieved control of their eating disorders, through the testimonies of patients commenting on the positive results they have obtained. These testimonies are included in the news article that complements the video. An example of these testimonies is as follows:

Pierre Vial has lost 43 kilos over the past nine and a half months. He and other patients at the eating disorder clinic explain the effects obesity and anorexia have had on their lives. Another patient, Karin Borell, still has some months to go at the clinic but, after decades of battling anorexia, is beginning to be able to visualise life without the illness: “On a good day I see myself living a normal life without an eating disorder, without problems with food. That’s really all I wish right now”.[32]

This qualitative evidence shows how the research results contribute to the achievement of the target goals of the call addressed:“ICT-2013.5.1—Personalised health, active ageing, and independent living”. [33] In this case, the results are robust, particularly for people suffering chronic diseases and desiring to improve their health; people who have applied the research findings are improving their eating disorders and better managing their health. The value of this evidence is the inclusion of the patients’ voices stating the impact of the research results on their health.

The second example is a Facebook post from project 9, which provides a link to a Euronews video. The aim of this project is to bring some tools from the lab to the farm in order to guarantee a better management of the farm and animal welfare. In this video [34], there are quotes from farmers using the new system developed through the research results of the project. These quotes show how use of the new system is improving the management of the farm and the health of the animals; some examples are provided:

Cameras and microphones help me detect in real time when the animals are stressed for whatever reason,” explained farmer Twan Colberts. “So I can find solutions faster and in more efficient ways, without me being constantly here, checking each animal.”

This evidence shows how the research results contribute to addressing the objectives specified in the call “KBBE.2012.1.1–02—Animal and farm-centric approach to precision livestock farming in Europe” [29], particularly, to improve the precision of livestock farming in Europe. The interaction obtained is composed of6 likes and 1 share. The profiles are diverse, but some of them do not disclose personal information; others have not added a profile description, and only their name and photo are available.

### Interrater reliability (kappa)

The analysis of tweets and Facebook posts providing linkages with information about social impact was conducted following a content analysis method in which reliability was based on a peer review process. This sample is composed of 3,425 tweets and 1,925 Fb/posts. Each tweet and Facebook post was analysed to identify whether or not it contains evidence of social impact. Each researcher has the codebook a priori. We used interrater reliability in examining the agreement between the two raters on the assignment of the categories defined through Cohen’s kappa. We used SPSS to calculate this coefficient. We exported an excel sheet with the sample coded by the two researchers being 1 (is evidence of social impact, either potential or real) and 0 (is not evidence of social impact) to SPSS. The cases where agreement was not achieved were not considered as containing evidence of social impact. The result obtained is 0.979; considering the interpretation of this number according to Landis & Koch [35], our level of agreement is almost perfect, and thus, our analysis is reliable. To sum up the data analysis, the description of the steps followed is explained:

Step 1. Data analysis I. We included all data collected in an excel sheet to proceed with the analysis. Prior to the analysis, researchers read the codebook to keep in mind the information that should be identified.

Step 2. Each researcher involved reviewed case by case the tweets and Facebook posts to identify whether they provide links with evidence of social impact or not. If the researcher considers there to be evidence of social impact, he or she introduces the value of 1into the column, and if not, the value of 0.

Step 3. Once all the researchers have finished this step, the next step is to export the excel sheet to SPSS to extract the kappa coefficient.

Step 4. Data Analysis II. The following step was to analyse case by case the tweets and Facebook posts identified as providing linkages with information of social impact and classify them as quantitative or qualitative evidence of social impact.

Step 5. The interaction received was analysed because this determines to which extent this evidence of social impact has captured the attention of citizens (in the form of how many likes, shares, or retweets the post has).

Step 6. Finally, if available, the profile descriptions of the citizens interacting through retweeting or sharing the Facebook post were considered.

Step 7. SICOR was calculated. It could be applied to the complete sample (all data projects) or to each project, as we will see in the next section.

## Results

The total number of tweets and Fb/posts collected from the 10 projects is 5,350. After the content analysis, we identified 23 tweets and Facebook posts providing linkages to information about social impact. To respond to the research question, which considered whether there is evidence of social impact shared by citizens in social media, the answer was affirmative, although the coverage ratio is low. Both Twitter and Facebook users retweeted or shared evidence of social impact, and therefore, these two social media networks are valid sources for expanding knowledge on the assessment of social impact. Table 10 shows the social impact coverage ratio in relation to the total number of messages analysed.

**Table 10:** Relation of tweets/Fb posts with evidence of social impact.

Total tweets/ Fb posts	5,350
Total tweets/ Fb posts with evidence	23
Social Impact Coverage Ratio	0,43%

The analysis of each of the projects selected revealed some results to consider. Of the 10 projects, 7 had evidence, but those projects did not necessarily have more Tweets and Facebook posts. In fact, some projects with fewer than 70 tweets and 50 Facebook posts have more evidence of social impact than other projects with more than 400 tweets and 400 Facebook posts. This result indicates that the number of tweets and Facebook posts does not determine the existence of evidence of social impact in social media. For example, project 2 has 403 tweets and 423 Facebooks posts, but it has no evidence of social impact on social media. In contrast, project 9 has 62 tweets, 43 Facebook posts, and 2 pieces of evidence of social impact in social media, as shown in Table 11.

**Table 11:** Total number of tweets and Facebook posts with evidence of social impact.

Project	Tweets	Tweets with evidence of potential/real social impact	Facebook posts	Facebook posts with evidence of potential/real social impact
Project 1	965	2	585	0
Project 2	403	0	423	0
Project 3	903	0	396	1
Project 4	26	2	41	1
Project 5	418	1	16	0
Project 6	302	0	74	0
Project 7	157	6	64	5
Project 8	65	0	236	0
Project 9	62	1	43	1
Project 10	124	3	47	0
TOTAL	3,425	15	1,925	8

The ratio of tweets/Fb posts to evidence is 0.43%, and it differs depending on the project, as shown below in Table 12. There is one project (P7) with a ratio of 4.98%, which is a social impact coverage ratio higher than that of the other projects. Next, a group of projects (P3, P9, P10) has a social impact coverage ratio between 1.41% and 2,99%.The next slot has three projects (P1, P4, P5), with a ratio between 0.13% and 0.46%. Finally, there are three projects (P2, P6, P8) without any tweets/Fb posts evidence of social impact.

**Table 12:** Social impact coverage ratio per project.

Projects	Total tweets/ Fb posts	Total tweets/ Fb posts with potential/real social impact	Social Impact Coverage Ratio
Project 1	1,550	2	0,13%
Project 2	826	0	0,00
Project 3	67	2	2,99%
Project 4	434	2	0,46%
Project 5	376	1	0,27%
Project 6	376	0	0,00
Project 7	221	11	4,98%
Project 8	301	0	0,00
Project 9	105	2	1,90%
Project 10	171	3	1,75%

Considering the three strategies for obtaining data, each is related differently to the evidence of social impact. In terms of the social impact coverage ratio, as shown in Table 13, the most successful strategy is number 3 (searchable research results), as it has a relation of 17.86%, which is much higher than the ratios for the other 2 strategies. The second strategy (acronym search) is more effective than the first (profile accounts),with 1.77% for the former as opposed to 0.27% for the latter.

**Table 13:** Social impact coverage ratio per search strategy.

	Total tweets/ Fb posts	Total tweets/ Fb posts with potential/real social impact	Social Impact Coverage Ratio
Strategy 1 (profile accounts)	5,096	14	0,27%
Strategy 2 (acronym search)	226	4	1,77%
Strategy 3 (searchable research results)	28	5	17,86%
Total	5,350	23	

Once tweets and Facebook posts providing linkages with information about social impact(ESISM)were identified, we classified them in terms of quantitative (QUANESISM) or qualitative evidence (QUALESISM)to determine which type of evidence was shared in social media. Table 14 indicates the amount of quantitative and qualitative evidence identified for each search strategy.

**Table 14:** Amount of each type of evidence for each search strategy.

Strategy 1				Strategy 2		Strategy 3	
Profile twitter		Facebook page		Acronym Search (Twitter)		Searchable Research Result (Twitter)	
ESISM-QUANESISM	ESISM QUALESISM	ESISM QUANESISM	ESISM QUALESISM	ESISM QUANESISM	ESISM QUALESISM	ESISM QUANESIM	ESISM QUALESISM
3	3	6	2	1	3	4	1

## Discussion

First, the results obtained indicated that the SISM methodology aids in calculating the social impact coverage ratio of the research projects selected and evaluating whether the social impact of the corresponding research is shared by citizens in social media. The social impact coverage ratio applied to the sample selected is low, but when we analyse the SICOR of each project separately, we can observe that some projects have a higher social impact coverage ratio than others. Complementary to altmetrics measuring the extent to which research results reach out society, the SICOR considers the question whether this process includes evidence of potential or real social impact. In this sense, the overall methodology of SISM contributes to advancement in the evaluation of the social impact of research by providing a more precise approach to what we are evaluating.

This contribution complements current evaluation methodologies of social impact that consider which improvements are shared by citizens in social media. Exploring the results in more depth, it is relevant to highlight that of the ten projects selected, there is one research project with a social impact coverage ratio higher than those of the others, which include projects without any tweets or Facebook posts with evidence of social impact. This project has a higher ratio of evidence than the others because evidence of its social impact is shared more than is that of other projects. This also means that the researchers produced evidence of social impact and shared it during the project. Another relevant result is that the quantity of tweets and Fb/posts collected did not determine the number of tweets and Fb/posts found with evidence of social impact. Moreover, the analysis of the research projects selected showed that there are projects with less social media interaction but with more tweets and Fb/posts containing evidence of social media impact. Thus, the number of tweets and Fb/posts with evidence of social impact is not determined by the number of publication messages collected; it is determined by the type of messages published and shared, that is, whether they contain evidence of social impact or not.

The second main finding is related to the effectiveness of the search strategies defined. Related to the strategies carried out under this methodology, one of the results found is that the most effective search strategy is the searchable research results, which reveals a higher percentage of evidence of social impact than the own account and acronym search strategies. However, the use of these three search strategies is highly recommended because the combination of all of them makes it possible to identify more tweets and Facebook posts with evidence of social impact.

Another result is related to the type of evidence of social impact found. There is both quantitative and qualitative evidence. Both types are useful for understanding the type of social impact achieved by the corresponding research project. In this sense, quantitative evidence allows us to understand the improvements obtained by the implementation of the research results and capture their impact. In contrast, qualitative evidence allows us to deeply understand how the resultant improvements obtained from the implementation of the research results are evaluated by the end users by capturing their corresponding direct quotes. The social impact includes the identification of both real and potential social impact.

## Conclusions

After discussing the main results obtained, we conclude with the following points. Our study indicates that there is incipient evidence of social impact, both potential and real, in social media. This demonstrates that researchers from different fields, in the present case involved in medical research, public health, animal welfare and genomics, are sharing the improvements generated by their research and opening up new venues for citizens to interact with their work. This would imply that scientists are promoting not only the dissemination of their research results but also the evidence on how their results may lead to the improvement of societies. Considering the increasing relevance and presence of the dissemination of research, the results indicate that scientists still need to include in their dissemination and communication strategies the aim of sharing the social impact of their results. This implies the publication of concrete qualitative or quantitative evidence of the social impact obtained. Because of the inclusion of this strategy, citizens will pay more attention to the content published in social media because they are interested in knowing how science can contribute to improving their living conditions and in accessing crucial information. Sharing social impact in social media facilitates access to citizens of different ages, genders, cultural backgrounds and education levels. However, what is most relevant for our argument here is how citizens should also be able to participate in the evaluation of the social impact of research, with social media a great source to reinforce this democratization process. This contributes not only to greatly improving the social impact assessment, as in addition to experts, policy makers and scientific publications, citizens through social media contribute to making this assessment much more accurate. Thus, citizens’ contribution to the dissemination of evidence of the social impact of research yields access to more diverse sectors of society and information that might be unknown by the research or political community. Two future steps are opened here. On the one hand, it is necessary to further examine the profiles of users who interact with this evidence of social impact considering the limitations of the privacy and availability of profile information. A second future task is to advance in the articulation of the role played by citizens’ participation in social impact assessment, as citizens can contribute to current worldwide efforts by shedding new light on this process of social impact assessment and contributing to making science more relevant and useful for the most urgent and poignant social needs.

## Supporting information

S1 File Interrater reliability (kappa) result. This file contains the SPSS file with the result of the calculation of Cohen’s Kappa regards the interrater reliability. The word document exported with the obtained result is also included.
(ZIP)

S2 File Data collected and SICOR calculation. This excel contains four sheets, the first one titled “data collected” contains the number of tweets and Facebook posts collected through the three defined search strategies; the second sheet titled “sample” contains the sample classified by project indicating the ID of the message or code assigned, the type of message (tweet or Facebook post) and the codification done by researchers being 1 (is evidence of social impact, either potential or real) and 0 (is not evidence of social impact); the third sheet titled “evidence found” contains the number of type of evidences of social impact founded by project (ESISM-QUANESIM or ESISM-QUALESIM), search strategy and type of message (tweet or Facebook posts); and the last sheet titled “SICOR” contains the Social Impact Coverage Ratio calculation by projects in one table and type of search strategy done in another one.
(XLSX)
