# Supplementary material for: Social impact in social media: A new method to evaluate the social impact of research
Source: PLoS One. 2018 Aug 29;13(8):e0203117. doi: 10.1371/journal.pone.0203117 (PMC6114920; doi:10.1371/journal.pone.0203117)
Supplement: S1 File — This file contains the SPSS file with the result of the calculation of Cohen’s Kappa regards the interrater reliability. The word document exported with the obtained result is also included. (ZIP) [file pone.0203117.s001.zip › S1_Kappa/KAPPA RESULT.doc]

SET DIGITGROUPING=No Small=0.0001 Unicode=Yes OLang=English.
FREQUENCIES VARIABLES=RESEARCHER1 RESEARCHER2
  /ORDER=ANALYSIS.


Frequencies


Notes	
Output Created	05-APR-2018 14:43:25	
Comments		
Input	Data	D:\Usuarios\usuari\Desktop\datos.sav	
	Active Dataset	DataSet1	
	Filter	<none>	
	Weight	<none>	
	Split File	<none>	
	N of Rows in Working Data File	5350	
Missing Value Handling	Definition of Missing	User-defined missing values are treated as missing.	
	Cases Used	Statistics are based on all cases with valid data.	
Syntax	FREQUENCIES VARIABLES=RESEARCHER1 RESEARCHER2
  /ORDER=ANALYSIS.	
Resources	Processor Time	00:00:00,00	
	Elapsed Time	00:00:00,01	


[DataSet1] D:\Usuarios\usuari\Desktop\datos.sav


Statistics	
	Researcher 1 codes	Researcher 2 codes	
N	Valid	5350	5350	
	Missing	0	0	


Frequency Table


Researcher 1 codes	
	Frequency	Percent	Valid Percent	Cumulative Percent	
Valid	Without social impact	5326	99,6	99,6	99,6	
	With social impact	24	,4	,4	100,0	
	Total	5350	100,0	100,0		


Researcher 2 codes	
	Frequency	Percent	Valid Percent	Cumulative Percent	
Valid	Sin impacto social	5327	99,6	99,6	99,6	
	Con impacto social	23	,4	,4	100,0	
	Total	5350	100,0	100,0		

RELIABILITY
  /VARIABLES=RESEARCHER1 RESEARCHER2
  /SCALE('ALL VARIABLES') ALL
  /MODEL=ALPHA
  /STATISTICS=DESCRIPTIVE SCALE HOTELLING CORR ANOVA TUKEY
  /SUMMARY=TOTAL CORR
  /ICC=MODEL(MIXED) TYPE(CONSISTENCY) CIN=95 TESTVAL=0.


Reliability


Notes	
Output Created	05-APR-2018 14:44:29	
Comments		
Input	Data	D:\Usuarios\usuari\Desktop\datos.sav	
	Active Dataset	DataSet1	
	Filter	<none>	
	Weight	<none>	
	Split File	<none>	
	N of Rows in Working Data File	5350	
	Matrix Input		
Missing Value Handling	Definition of Missing	User-defined missing values are treated as missing.	
	Cases Used	Statistics are based on all cases with valid data for all variables in the procedure.	
Syntax	RELIABILITY
  /VARIABLES=RESEARCHER1 RESEARCHER2
  /SCALE('ALL VARIABLES') ALL
  /MODEL=ALPHA
  /STATISTICS=DESCRIPTIVE SCALE HOTELLING CORR ANOVA TUKEY
  /SUMMARY=TOTAL CORR
  /ICC=MODEL(MIXED) TYPE(CONSISTENCY) CIN=95 TESTVAL=0.	
Resources	Processor Time	00:00:00,02	
	Elapsed Time	00:00:00,02	


[DataSet1] D:\Usuarios\usuari\Desktop\datos.sav


Scale: ALL VARIABLES


Case Processing Summary	
	N	%	
Cases	Valid	5350	100,0	
	Excludeda	0	,0	
	Total	5350	100,0	

a. Listwise deletion based on all variables in the procedure.	


Reliability Statistics	
Cronbach's Alpha	Cronbach's Alpha Based on Standardized Items	N of Items	
,989	,989	2	


Item Statistics	
	Mean	Std. Deviation	N	
Researcher 1 codes	,00	,067	5350	
Researcher 2 codes	,00	,065	5350	


Inter-Item Correlation Matrix	
	Researcher 1 codes	Researcher 2 codes	
Researcher 1 codes	1,000	,979	
Researcher 2 codes	,979	1,000	


Summary Item Statistics	
	Mean	Minimum	Maximum	Range	Maximum / Minimum	Variance	
Inter-Item Correlations	,979	,979	,979	,000	1,000	,000	

Summary Item Statistics	
	N of Items	
Inter-Item Correlations	2	


Item-Total Statistics	
	Scale Mean if Item Deleted	Scale Variance if Item Deleted	Corrected Item-Total Correlation	Squared Multiple Correlation	
Researcher 1 codes	,00	,004	,979	,958	
Researcher 2 codes	,00	,004	,979	,958	

Item-Total Statistics	
	Cronbach's Alpha if Item Deleted	
Researcher 1 codes	.	
Researcher 2 codes	.	


Scale Statistics	
Mean	Variance	Std. Deviation	N of Items	
,01	,017	,132	2	


ANOVA with Tukey's Test for Nonadditivity	
	Sum of Squares	df	Mean Square	F	
Between People	46,294	5349	,009		
Within People	Between Items	,000	1	,000	1,000	
	Residual	Nonadditivity	,005a	1	,005	57,371	
		Balance	,495	5348	,000		
		Total	,500	5349	,000		
	Total	,500	5350	,000		
Total	46,794	10699	,004		

ANOVA with Tukey's Test for Nonadditivity	
	Sig	
Between People		
Within People	Between Items	,317	
	Residual	Nonadditivity	,000a	
		Balance		
		Total		
	Total		
Total		

Grand Mean = ,00	
a. Tukey's estimate of power to which observations must be raised to achieve additivity = ,497.	


Hotelling's T-Squared Test	
Hotelling's T-Squared	F	df1	df2	Sig	
1,000	1,000	1	5349	,317	


Intraclass Correlation Coefficient	
	Intraclass Correlationb	95% Confidence Interval	F Test with True Value 0	
		Lower Bound	Upper Bound	Value	df1	df2	
Single Measures	,979a	,977	,980	92,604	5349	5349	
Average Measures	,989c	,989	,990	92,604	5349	5349	

Intraclass Correlation Coefficient	
	F Test with True Value 0b	
	Sig	
Single Measures	,000a	
Average Measures	,000c	

Two-way mixed effects model where people effects are random and measures effects are fixed.	
a. The estimator is the same, whether the interaction effect is present or not.	
b. Type C intraclass correlation coefficients using a consistency definition-the between-measure variance is excluded from the denominator variance.	
c. This estimate is computed assuming the interaction effect is absent, because it is not estimable otherwise.	

CROSSTABS
  /TABLES=RESEARCHER1 BY RESEARCHER2
  /FORMAT=AVALUE TABLES
  /STATISTICS=CHISQ CORR KAPPA
  /CELLS=COUNT
  /COUNT ROUND CELL.


Crosstabs


Notes	
Output Created	05-APR-2018 14:46:08	
Comments		
Input	Data	D:\Usuarios\usuari\Desktop\datos.sav	
	Active Dataset	DataSet1	
	Filter	<none>	
	Weight	<none>	
	Split File	<none>	
	N of Rows in Working Data File	5350	
Missing Value Handling	Definition of Missing	User-defined missing values are treated as missing.	
	Cases Used	Statistics for each table are based on all the cases with valid data in the specified range(s) for all variables in each table.	
Syntax	CROSSTABS
  /TABLES=RESEARCHER1 BY RESEARCHER2
  /FORMAT=AVALUE TABLES
  /STATISTICS=CHISQ CORR KAPPA
  /CELLS=COUNT
  /COUNT ROUND CELL.	
Resources	Processor Time	00:00:00,02	
	Elapsed Time	00:00:00,03	
	Dimensions Requested	2	
	Cells Available	174734	


[DataSet1] D:\Usuarios\usuari\Desktop\datos.sav


Case Processing Summary	
	Cases	
	Valid	Missing	Total	
	N	Percent	N	Percent	N	Percent	
Researcher 1 codes * Researcher 2 codes	5350	100,0%	0	0,0%	5350	100,0%	


Researcher 1 codes * Researcher 2 codes Crosstabulation	
Count  	
	Researcher 2 codes	Total	
	Sin impacto social	Con impacto social		
Researcher 1 codes	Without social impact	5326	0	5326	
	With social impact	1	23	24	
Total	5327	23	5350	


Chi-Square Tests	
	Value	df	Asymp. Sig. (2-sided)	Exact Sig. (2-sided)	Exact Sig. (1-sided)	
Pearson Chi-Square	5126,121a	1	,000			
Continuity Correctionb	4904,686	1	,000			
Likelihood Ratio	288,258	1	,000			
Fisher's Exact Test				,000	,000	
Linear-by-Linear Association	5125,163	1	,000			
N of Valid Cases	5350					

a. 1 cells (25,0%) have expected count less than 5. The minimum expected count is ,10.	
b. Computed only for a 2x2 table	


Symmetric Measures	
	Value	Asymp. Std. Errora	Approx. Tb	
Interval by Interval	Pearson's R	,979	,021	349,932	
Ordinal by Ordinal	Spearman Correlation	,979	,021	349,932	
Measure of Agreement	Kappa	,979	,021	71,597	
N of Valid Cases	5350			

Symmetric Measures	
	Approx. Sig.	
Interval by Interval	Pearson's R	,000	
Ordinal by Ordinal	Spearman Correlation	,000	
Measure of Agreement	Kappa	,000	
N of Valid Cases		

a. Not assuming the null hypothesis.	
b. Using the asymptotic standard error assuming the null hypothesis.	
c. Based on normal approximation.	
